# Hemophagocytic Lymphohistiocytosis (HLH): A Rare Complication of Dengue Hemorrhagic Fever

**DOI:** 10.7759/cureus.70895

**Published:** 2024-10-05

**Authors:** Muhammad Raza, Shehrbano Ali

**Affiliations:** 1 Internal Medicine, Combined Military Hospital (CMH) Lahore Medical College and Institute of Dentistry, Lahore, PAK; 2 Internal Medicine, Combined Military Hospital, Lahore, PAK; 3 Medical School, Combined Military Hospital (CMH) Lahore Medical College and Institute of Dentistry, Lahore, PAK

**Keywords:** dengue-associated hlh, dengue fever/complications, dengue hemorrhagic fever, hemophagocytic lymphohistiocytosis (hlh), secondary hemophagocytic lymphohistiocytosis (hlh)

## Abstract

Dengue-associated hemophagocytic lymphohistiocytosis (HLH) is a rare presentation that can be potentially life-threatening, characterized by excessive activation of the immune system. This case report highlights the complexity of the presentation and management of a patient with multiple comorbidities who developed severe complications following a diagnosis of dengue fever. The patient presented with a persistent fever and abdominal pain alongside worsening renal and liver function. The combination of elevated serum triglyceride and ferritin levels, along with bicytopenia and the presence of splenomegaly, eventually led to a diagnosis of HLH. The patient required intensive care support, including hemodialysis, blood transfusions, and oxygen therapy. Treatment for HLH was initiated with high-dose dexamethasone and plasmapheresis, aiming to control the overactive immune response. This case highlights the need for awareness of HLH as a complication of dengue fever and emphasizes the significance of early diagnosis and treatment. It also stresses the need for further research to improve diagnostic and management strategies for HLH in dengue-endemic regions.

## Introduction

Hemophagocytic lymphocytosis (HLH) is a considerably rare, life-threatening disorder characterized by an overactive immune system that causes severe inflammation leading to multi-organ failure [1]. The pathogenesis underlying this phenomenon is the inappropriate activation of natural killer (NK) cells and cytotoxic T lymphocytes, which leads to an uncontrolled release of interleukins and cytokines, resulting in the cytokine release storm [2].

HLH can be divided into two types. The first is primary (familial) HLH, which is more common in children and linked to genetic mutations. The second is secondary HLH, which is caused by an acquired immune system overdrive in response to infection, malignancy, or autoimmune disease [3]. Some bacterial, fungal, and parasitic infections have been associated with secondary HLH, but viral infections are the most common cause under the category of infections. Among the reported viral causes associated with HLH are the Epstein-Barr virus, cytomegalovirus, parvovirus, herpes viruses, HIV, influenza, and hepatitis viruses [4].

Dengue fever is an epidemic rampant in African and Southeast Asian countries, particularly during the monsoon season. The disease, caused by a flavivirus, can vary in intensity, ranging from mild dengue fever to severe forms such as dengue hemorrhagic fever (DHF) and dengue shock syndrome (DSS) [5]. Dengue-associated HLH is a severe complication of dengue fever that remains poorly defined, currently understood through case reports, with no large-scale studies present. Patients with dengue-associated HLH often present with varying symptoms, complicating diagnosis due to significant clinical overlap with DHF, both of which present with bleeding diathesis, prompting the need for large-scale research [6].

## Case presentation

A 72-year-old male, known to have diabetes mellitus, hypertension, and benign prostatic hyperplasia (BPH), presented to the tertiary care hospital with a high-grade fever that had persisted for the last five days. The fever was accompanied by rigors, chills, and generalized body aches. The patient also reported decreased appetite and periumbilical, non-radiating abdominal pain that started with the fever. Additionally, he experienced four episodes of vomiting, which was non-bilious, starting a day prior to presentation at the hospital. The patient also mentioned a history of constipation for the last eight days. There was no history of smoking or alcohol intake. Prior to this presentation at the hospital, the patient had already been tested for dengue virus via a non-structural protein 1 (NS1) antigen test, which had returned positive. Given the clinical presentation and positive NS1 antigen test, a diagnosis of dengue fever with possible complications was considered.

The patient was drowsy on presentation but responsive to commands, with normal plantar reflexes and pupils that were equal and reactive. Jaundice was noted upon examination. Some tenderness and mild distension were observed on abdominal examination, but there was no evidence of hepatomegaly or splenomegaly. The cardiothoracic exam showed no abnormalities, but the chest exam detected bilateral coarse crepitations. Blood pressure was measured at 170/70 mmHg, with a pulse rate of 88 beats per minute. The patient was admitted to the High Dependency Unit (HDU) for close monitoring and management.

The initial laboratory investigations revealed significant abnormalities. Complete blood counts indicated anemia with a hemoglobin (Hb) level of 10.9 g/dL, low red blood cells (RBCs) at 3.45 x 10^12^/L, thrombocytopenia with low platelets at 40 x 10^9^/L, and an elevated total leukocyte count (TLC) at 14.8 x 10^9^/L. C-reactive protein (CRP) was raised at 110 mg/L. Liver function tests were notably deranged, with total bilirubin at 154 µmol/L, alkaline phosphatase (ALP) at 236 U/L, alanine aminotransferase (ALT) at 3074 U/L, and albumin at 34 g/L. Kidney function tests also showed severe abnormalities, with urea at 23.1 mmol/L and serum creatinine at 432 µmol/L, pointing towards evidence of acute kidney as well as liver injury. Table 1 shows the baseline laboratory results over the course of admission.

**Table 1:** Laboratory results over the course of admission TLC: total leukocyte count; RBC: red blood cells; Hb: hemoglobin; ALP: alkaline phosphatase; ALT: alanine aminotransferase

Parameters	Reference Range	Day 1	Day 2	Day 3
TLC	4-10 x 10^9^/L	14.8	13.1	9.4
RBC	4.5-6.5 x 10^12^/L	3.45	3.64	4.46
Hb	13.5-17.5 g/dL	10.9	11.5	11.6
Platelet count	150-400 x 10^9^/L	40	46	60
CRP (quantitative)	< 6 mg/L	110	123.9	-
Serum urea	1.8-7.1 mmol/L	23.1	34.2	-
Serum creatinine	62-120 µmol/L	432	735	553
Serum sodium	135-150 mmol/L	133	132	134
Serum potassium	3.5-5.0 mmol/L	3.6	4.5	4.9
Total bilirubin	0-17 µmol/L	154	222	311
ALP	42-141 U/L	236	285	243
ALT	0-59 U/L	3074	2081	1363
Albumin	35-50 g/L	34	30	-

After admission, the patient was started on laxatives, calcium acetate (a phosphate binder), and sodium bicarbonate for metabolic management. Antidiabetic and antihypertensive medications were optimized for the duration of hospital stay, and intravenous fluids were initiated. Antibiotic coverage via piperacillin/tazobactam 4.5 g BD was also started. Careful input and output charting was also advised, along with close monitoring of vital signs, neurology, and any source of bleeding. Over the first 24 hours of admission, the patient's condition worsened with deteriorating oxygen saturation on room air and worsening neurology. Oxygen support was started, and the patient was transferred to the intensive care unit (ICU).

In light of the kidney and liver injuries, respectively, hemodialysis for three consecutive days was advised, and the patient was started on lactulose, rifaximin, and HepaMerz for the management of hepatic encephalopathy. In order to carry out hemodialysis, venous access was required, for which a double-lumen catheter was passed in the right femoral vein of the patient. However, during the procedure, excessive blood loss was observed, pointing toward the development of a bleeding diathesis.

Further investigations revealed highly elevated serum ferritin levels (>2000 ng/mL), a significantly increased lactate dehydrogenase (LDH) level of 6054 U/L, and an elevated pro-BNP level of 12,021 pg/mL. Cardiac enzymes showed a creatine kinase (CK) level of 3031 U/L. The plasma fibrinogen level was found to be slightly below normal. Ultrasound of the abdomen revealed mild splenomegaly. Table 2 provides the results of detailed investigations ordered during the course of the hospital stay.

**Table 2:** Results of detailed laboratory investigations ordered during hospital stay LDH: lactate dehydrogenase; pro-BNP: pro-B-type natriuretic peptide; PTTK: partial thromboplastin time; INR: international normalized ratio; CK: creatine kinase; AST: aspartate aminotransferase; pH: potential of hydrogen; pCO_2_: partial pressure of carbon dioxide; pO_2_: partial pressure of oxygen

Parameters	Reference Range	Results
Serum ferritin	< 500 µg/L	> 2000
Serum LDH	140-280 U/L	6054
Serum triglycerides	< 1.7 mmol/L	2.8
Serum lactate	< 2 mmol/L	8.7
Pro-BNP	< 125 pg/mL	12021
Coagulation profile		
PTTK	25-35 seconds	35
INR	< 1.1	1.0
Plasma fibrinogen	200-400 mg/dL	198
Cardiac enzymes		
Serum CK	Up to 192 U/L	3031
Serum AST	< 37 U/L	5
Serum LDH	135-225 U/L	4407
Bone profile		
Serum phosphate	0.81-1.5 mmol/L	1.3
Serum calcium total	2.1-2.65 mmol/L	2.1
Serum magnesium	0.6-1.0 mmol/L	0.8
Arterial blood gases		
pH	7.35-7.45	7.47
pCO_2_	35-45 mmHg	23.3
pO_2_	80-110 mmHg	233
Bicarbonate	22-28 mmol/L	17.3
Base excess	-3 to +3 mmol/L	-6.2

Two important differential diagnoses considered in this case were leptospirosis and thrombotic thrombocytopenic purpura (TTP). Leptospirosis is a bacterial infection that can present with similar clinical features, including fever, abdominal pain, and jaundice. In our case, leptospirosis was ruled out based on the patient's clinical symptoms and history. Despite the presence of factors common to both leptospirosis and HLH, including high white blood cell counts, elevated CRP levels, and renal and hepatic involvement, the patient did not show signs typically associated with leptospirosis, such as conjunctival suffusion, and there was no reported exposure to contaminated water. For TTP, while the patient had anemia and thrombocytopenia, further evaluation revealed no schistocytes in the blood smear, which are usually present in TTP cases. Furthermore, the positive NS1 antigen test for dengue fever and the fulfillment of six out of the eight HLH diagnostic criteria significantly pointed toward the diagnosis of HLH.

Based on persistent high-grade fever for more than seven days, elevated serum ferritin levels > 500 µg/L, a mild rise in triglyceride levels, low levels of fibrinogen, splenomegaly, and bicytopenia (anemia and thrombocytopenia), six out of the eight criteria for diagnosing HLH were satisfied, with an H-score of 225 points, giving a 96% probability of confirmation of HLH. The rapid deterioration of the patient's condition with abnormal liver and renal function and a recent history of dengue fever further supported the presence of an underlying complication. To further confirm the diagnosis, a bone marrow biopsy was done, which also revealed the presence of hemophagocytes, characterized by activated macrophages engulfing hematopoietic cells. The patient was started on high-dose dexamethasone to manage HLH, and plasmapheresis (PLEX) sessions were planned. However, the patient passed away prior to the initiation of the first PLEX session.

## Discussion

Our patient initially presented with a persistent fever five days after being diagnosed with dengue fever. As the condition worsened, the patient required oxygen support, intensive care, and hemodialysis. Laboratory investigations showed very high levels of ferritin, LDH, and triglycerides, along with abnormal liver and kidney function, as well as low blood cell counts. Combined with the recent history of dengue fever, this resulted in the diagnosis of HLH. The patient was started on high-dose dexamethasone, and plasmapheresis was planned before the patient expired.

HLH associated with the dengue virus is a significantly rare, life-threatening occurrence that results when the virus triggers the immune system to go into overdrive. This happens when regulatory pathways that normally control the immune response get overwhelmed. Excessive activation of macrophages and T cells leads to widespread inflammation, resulting in severe complications [7].

A systematic review of HLH associated with dengue fever reported a fatality rate of 14.6% among 122 reported cases. The time from admission to diagnosis ranged from one to 15 days, with an average of approximately five days (mean: 4.97; SD: 3.86). One case was presumed to be dengue due to overlapping symptoms; it was identified to be HLH later on autopsy. The study found that 97.2% of cases had a fever, 70.2% had hepatomegaly, 78.4% had splenomegaly, 90.1% had thrombocytopenia, 76% had anemia, and 97.1% presented with serum ferritin levels > 500 μg/L [8]. Our case report aligns with most of the commonly reported findings in this review.

Diagnostic guidelines for HLH were initially established in 1991, requiring the fulfillment of all five criteria: fever, splenomegaly, bicytopenia, evidence of hemophagocytosis, and at least one of hypertriglyceridemia or hypofibrinogenemia [9]. HLH often presents in atypical ways; hence, guidelines were revised following the HLH trial in 2004. Three additional points were added to the previously existing five: low or absent NK cell activity, serum ferritin levels above 500 µg/L, and soluble CD25 (IL-2 receptor) levels of 2,400 U/mL or higher. According to recent guidelines, any five of these eight criteria must be satisfied to confirm a diagnosis of HLH [10].

The diagnosis of HLH is often delayed due to the rarity of the disease, variability in presentation, and complexity of diagnostic criteria; delay in diagnosis can have detrimental effects on the prognosis of this condition. Current diagnostic criteria for HLH are based on the few available studies and reported cases that overlook certain features, such as liver inflammation, neurological findings, and skin manifestations, which can present with HLH [11].

The 2004 treatment guidelines for HLH focus on aggressive therapy to control the disease. Initial treatment involves the use of etoposide, dexamethasone, and cyclosporin A. Intrathecal therapy with methotrexate and corticosteroids is recommended in case of central nervous system involvement. Close monitoring is essential to detect disease recurrence or progression. Reactivation of HLH may occur; hence, continuous therapy and treatment intensification may be required in that case. Hematopoietic stem cell transplantation is also recommended as early as possible for a potential cure for those with genetic or familial HLH or persistent disease [10].

A study focused on dengue-associated HLH found that timely treatment in such cases was of utmost importance due to high mortality. Initial treatment with corticosteroids was found to improve survival in every eight out of 10 patients; steroids help decrease inflammation due to immune system overactivity. Etoposide is a chemotherapeutic drug that was also shown to improve prognosis; this drug works by reducing activated T cells, which are seen as a result of exaggerated immune responses in HLH [12]. Chung et al. studied 16 reported cases of dengue-associated HLH, where 15 required corticosteroids and seven were given intravenous immunoglobulins (IVIG) to resolve symptoms [6].

Plasma exchange has shown potential benefits in reducing cytokine levels and improving clinical outcomes in severe HLH cases [13]. In a study involving 23 patients with secondary HLH, plasma exchange helped improve platelet counts alongside a reduction in serum ferritin and lactate dehydrogenase levels. Patients who received PLEX along with steroids had lower mortality rates compared to those managed with chemotherapy or steroids. This study reported that plasma exchange improved overall survival when used in combination with other treatment options [14].

This case highlights the significance of considering HLH as a potential complication in patients diagnosed with dengue fever in the presence of rapid deterioration and abnormal laboratory markers. Additionally, it contributes to the limited literature on dengue-associated HLH, particularly in adult patients with multiple comorbidities. The late presentation and diagnosis resulted in delayed treatment, which worsened the prognosis. The inability to test for NK cell activity and soluble CD25 levels could have provided more evidence to support the diagnosis.

Primary research and studies with large sample sizes are needed to better identify and manage secondary HLH. This will enhance our understanding of the disease course and prognosis and help improve treatment guidelines. Future trials should focus on improving diagnostic methods, particularly in dengue-endemic areas, to reduce mortality. Advancements in early diagnostic techniques and novel treatment methods will contribute to more effective management of HLH.

## Conclusions

This case report highlights the importance of considering HLH as a potential complication in patients deteriorating after a diagnosis of dengue fever. The overlapping clinical features between DHF and HLH can make early diagnosis challenging, but quick recognition and intervention are vital to improve patient outcomes. Despite initial treatment efforts, the patient's condition rapidly worsened due to the complex interplay of dengue fever and HLH. HLH associated with dengue fever is challenging to diagnose and manage, with a very high mortality rate. Timely recognition and treatment are crucial, as delays can worsen outcomes. Better diagnostic tools and treatment options are essential to enhance patient survival and management in dengue-endemic regions.
